# Injuries of the Spine; with an Analysis of Nearly Four Hundred Cases

**Published:** 1867-05

**Authors:** 


Injuries of the Spine; with an Analysis of nearly four hundred cases. By John Ashhurst, Jr., A.M., M.D., Fellow of College of Physicians of Philadelphia, etc., etc., etc. Philadelphia: J. B. Lippincott & Co. 1867. pp. 127.

This is a well written and very valuable essay on a very important subject. We fully commend it to our readers as worthy of a careful perusal. For sale by S. C. Griggs & Co., 148 Lake St.

